# Transcriptome analysis of copper homeostasis genes reveals coordinated upregulation of *SLC31A1*,*SCO*1, and *COX11* in colorectal cancer

**DOI:** 10.1002/2211-5463.12060

**Published:** 2016-07-08

**Authors:** Vincenza Barresi, Angela Trovato‐Salinaro, Giorgia Spampinato, Nicolò Musso, Sergio Castorina, Enrico Rizzarelli, Daniele Filippo Condorelli

**Affiliations:** ^1^ Section of Medical BiochemistryDepartment of Biomedical and Biotechnological SciencesUniversity of CataniaItaly; ^2^ Section of Human AnatomyDepartment of Biomedical and Biotechnological SciencesUniversity of CataniaItaly; ^3^ Institute of Biostructures and BioimagingNational Council of ResearchUOS CataniaItaly

**Keywords:** alternative transcripts, colorectal cancer, copper homeostasis, copper transporter, synthesis of cytochrome c oxidase

## Abstract

Copper homeostasis and distribution is strictly regulated by a network of transporters and intracellular chaperones encoded by a group of genes collectively known as copper homeostasis genes (CHGs). In this work, analysis of The Cancer Genome Atlas database for somatic point mutations in colorectal cancer revealed that inactivating mutations are absent or extremely rare in CHGs. Using oligonucleotide microarrays, we found a strong increase in mRNA levels of the membrane copper transporter 1 protein [CTR1; encoded by the solute carrier family 31 member 1 gene (*SLC31A1* gene)] in our series of colorectal carcinoma samples. CTR1 is the main copper influx transporter and changes in its expression are able to induce modifications of cellular copper accumulation. The increased *SLC31A1* mRNA level is accompanied by a parallel increase in transcript levels for copper efflux pump ATP7A, copper metabolism Murr1 domain containing 1 (COMMD1), the cytochrome C oxidase assembly factors [synthesis of cytochrome c oxidase 1 (SCO1) and cytochrome c oxidase copper chaperone 11 (COX11)], the cupric reductase six transmembrane epithelial antigen of the prostate (STEAP3), and the metal‐regulatory transcription factors (MTF1, MTF2) and specificity protein 1 (SP1). The significant correlation between *SLC31A1*,*SCO1*, and *COX11* mRNA levels suggests that this transcriptional upregulation might be part of a coordinated program of gene regulation. Transcript‐level upregulation of *SLC31A1*,*SCO1*, and *COX11* was also confirmed by the analysis of different colon carcinoma cell lines (Caco‐2, HT116, HT29) and cancer cell lines of different tissue origin (MCF7, PC3). Finally, exon‐level expression analysis of *SLC31A1* reveals differential expression of alternative transcripts in colorectal cancer and normal colonic mucosa.

**Abbreviations**
ATOX1: antioxidant 1 copper chaperone
CCS: copper chaperone for SOD1
CHGs: copper homeostasis genes
COMMD1: copper metabolism Murr1 domain containing 1
COX11: cytochrome c oxidase copper chaperone 11
COX17: cytochrome c oxidase copper chaperone 17
CRC: colorectal cancer
CTR1: membrane copper transporter 1 protein
gDNA: genomic DNA
MSI: microsatellite instability
MTF1, MTF2: metal‐regulatory transcription factors
MTs: metallothioneins
PSR: probe selection region
RMA: robust multi‐array average
RPKM: reads per kilobase of transcript per million mapped reads
SCO1: synthesis of cytochrome c oxidase 1
SCO2: synthesis of cytochrome c oxidase 2
SD: standard deviation
SLC31A1: solute carrier family 31 member 1 gene (encoding CTR1 protein)
SP1: transcription factor or specificity protein 1
STEAP: six transmembrane epithelial antigen of the prostate
TAC: Affymetrix^®^ Transcriptome Analysis Console Software
TCGA: The Cancer Genome Atlas
TNM: tumor‐node‐metastasis

A classical approach in the search of anticancer therapies is based on the exploitation of quantitative and qualitative differences in biochemical processes between tumor and normal cells 1, 2, 3. Copper assumes a critical role in many biological processes, mainly as a catalytic cofactor of enzymes, 4 and its homeostasis is guaranteed by the activity of metal transporters and intracellular chaperones 5, 6, 7. In humans, disruption of this tightly regulated cellular copper homeostasis affects normal tissue development and a growing number of reports indicates an alteration of essential metal ion homeostasis (metallostasis) in tumors 8, 9.

The ‘membrane copper transporter 1’ (CTR1) protein, encoded by the *so**l**ute carrier family 31 member 1* (*SLC31A1*) gene, is generally considered as responsible for cellular copper uptake in all cells 10. Cu(I) is transported by CTR1 and reduction of extracellular Cu(II) to Cu(I) is catalyzed by a family of metalloreductases called ‘six transmembrane epithelial antigen of the prostate’ (STEAP). Four members of this protein family have been identified and it has been demonstrated that three of them, STEAP2, STEAP3, and STEAP4, are cupric reductases that stimulate copper cellular uptake 11.

The ‘antioxidant 1 copper chaperone’ (ATOX1) 12, the ‘copper chaperone for SOD1’ (CCS) 13, the ‘cytochrome c oxidase copper’ chaperones COX17 and COX11 14, 15, and the mitochondrial cytochrome c oxidase assembly proteins called ‘synthesis of cytochrome c oxidase’ (SCO1 and 2) 16 bind Cu(I) facilitating its transfer to specific cellular destinations. Copper is transferred by CCS to the cuproenzyme SOD1 or by SCO1, SCO2, COX11, and COX17 to cytochrome c oxidase. ATOX1 is responsible for the transfer of copper to exporting ATPase pumps (ATP7A/B”) 17, 18. ‘Copper metabolism Murr1 domain containing 1’ (COMMD1) is another protein that facilitates the efflux of copper probably via its interaction with the copper transporter ATP7B and ATP7A 19.

Copper can be detoxified by the metallothioneins (MTs), a family of small proteins (7 kDa) containing a high proportion of cysteins. Thiol groups of the MTs can bind metal ions with high affinity acting as a buffer system or a reservoir for essential copper and zinc ions 20.

In this study, we analyzed the profile of somatic mutations and the expression of the above‐mentioned genes involved in copper homeostasis (copper homeostasis genes: CHGs) in colorectal cancer (CRC) samples and normal colonic mucosae (Mu). Results show a series of significant changes in the expression levels of several CHGs that could be interpreted as the consequence of an intense activity of this functional network. In particular, a correlated upregulation of *SLC31A1*,* SCO1*, and *COX11* mRNA has been observed in cancer cells *in vivo* and in culture conditions.

## Materials and methods

### Patients

Twenty‐seven patients underwent surgical resection for primary invasive colorectal cancer at the ‘*Centro Clinico Diagnostico G.B. Morgagni*’ in Catania. Tumors were staged according to the tumor‐node‐metastasis (TNM) staging system of UICC**.** The mean age was 66.61 years (SD 16.31) for 18 male patients and 68.67 (SD 15.63) for nine female patients. The tumors were mainly in stage II or III. In 19 patients, a sample of the matched unaffected normal colonic mucosa was also taken at distance of 3–6 cm from the tumor. All CRC specimens were frozen and stored at −80 °C until to RNA extraction. Informed consent was obtained from all patients involved in this study. This project was approved by the Ethics Committee of ASL3 of Catania (Italy).

### RNA extraction

Total RNA was extracted from tissue and cell lines using RNeasy Mini Kit (Cat. No. 74104; Qiagen, Milan, Italy) according to the manufacturer's instructions. The concentration and the quality of the RNA were determined using a ND‐1000 spectrophotometer (NanoDrop, Thermo Scientific, Pero, Italy).

### Transcriptome analysis

Whole Transcript Expression analysis was performed using 100 ng of total RNA to produce amplified, and targets labeled in sense orientation for hybridization to the ‘GeneChip Human Transcriptome Array 2.0’ according to the protocol supplied by the manufacturer (Cat. No. 902310, Cat. No. 900720; Affymetrix UK Ltd., High Wycombe, UK). Human Transcriptome Array 2.0 contains > 6.0 million distinct probes for analyzing simultaneously 44 699 coding transcripts and 22 829 noncoding transcripts. Array scanning and data analysis were performed using affymetrix^®^ expression console^™^ software (v.1.4; Affymetrix UK Ltd.) that provides signal estimation and quality control functionality for the GeneChip^®^ Expression Arrays, and the affymetrix^®^ transcriptome analysis console (tac) Software (Affymetrix UK Ltd.), which performs statistical analysis and provides a list of differentially expressed genes. Expression level analysis was performed using the normalization method based on the processing algorithm called robust multi‐array average (*RMA*) 21.

The data have been deposited to public repository: ‘Gene Expression Omnibus‐GEO’ (www.ncbi.nlm.nih.gov/geo) and will be accessible through GEO: GSE73360.

### Cell cultures

Three human colorectal adenocarcinoma cell lines (Caco‐2, HT29, and HCT116) and two human noncolorectal adenocarcinoma cell lines (MCF7 and PC3) have been used in the present work. Human colon cancer Caco‐2 (ATCC number: HTB‐37) and HT29 (ATCC number: HTB‐38) cell lines were maintained in Dulbecco's Modified Eagle Medium (DMEM 1X; GIBCO, Cat No. 31965‐023 containing 4.5 g·L^−1^ of d‐glucose). The HCT116 (ATCC number: CCL‐247) cell line was cultured in McCoy 5A+ (McCoy's 5A Medium 1X GIBCO, Cat No. 36600021 containing 3 g·L^−1^ of d‐glucose), 1.5 mm l‐glutamine, and 2200 mg·L^−1^ of sodium bicarbonate. The human breast adenocarcinoma cell line MCF‐7 (ATCC number: HTB‐22) was maintained in DMEM medium (1X; GIBCO, Cat No. 31965‐023 containing 1 g·L^−1^ of d‐glucose). The human prostate adenocarcinoma cell line PC3 (ATCC number: CRL‐1435) cell line was grown in DMEM/F12 medium nutrient mixture (1X; GIBCO, Cat. No. 21331‐020) and 1.5 mm l‐glutamine. Each medium was supplemented with 10% FBS (Cat. No. 10270‐106; Life Technologies, Monza, Italy) and 100 U·mL^−1^ of penicillin–streptomycin (Cat. No 15140‐122; Life Technologies). The cell cultures were grown in flasks (75 cm^2^) and incubated at 37 °C in humidified atmosphere with 5% of CO_2_ and 95% of air. The culture medium was changed twice a week.

### MicroSatellite Instability analysis

Genomic DNA (gDNA) was extracted from each tissue using the DNeasy Blood & Tissue Kit (Cat. No. 69504; Qiagen) according to the manufacturer's instructions. The concentration and the quality of the DNA were determined using a ND‐1000 spectrophotometer (NanoDrop, Thermo Scientific).

Microsatellite instability (MSI) analysis was performed on matched samples of DNA (100 ng) obtained from CRC tumors and adjacent normal colonic tissue by fluorescent dye‐labeled PCR amplification using a panel of six microsatellite markers: four of them belonging to Bethesda panel (D2S123, D5S346, BAT25, and BAT26) together with two additional microsatellite markers (D17S250 and BAT40). PCR amplification was conducted using primer sequences previously reported by Dietmaier *et al*. 22.

5′‐Fluorescent dye‐labeled (NED for BAT25, VIC for BAT26, 6‐FAM for BAT40, PET for D5S346, FAM for D2S123, and VIC for D18S58) and unlabeled oligonucleotides were used for PCR reactions and the amplification products were analyzed by ABI PRISM 310 genetic analyzer using genescan analysis Software (Applied Biosystems, Monza, Italy).

International criteria for the determination of MSI in CRC were used to differentiate high instability (MSI‐H) from low instability (MSI‐L) or microsatellite stability (MSS). Tumors were defined as MSI‐H if two or more of the six microsatellite markers show instability; MSI‐L if only one of the six markers shows instability; and microsatellite stable (MSS) if none of the markers show instability. Since MSI‐L have clinical and biological feature similar to MSS tumors 23, they have been included in the MSS group in all the analysis. Therefore, in the rest of the paper, the abbreviation MSI has been used to indicate MSI‐H and the term MSS to indicate MSS and MSI‐L tumors. MSI status was successfully determined in all tumors and the analysis revealed that 22 tumors were MSS (8 females and 14 males) and five tumors were MSI (1 female and 4 males).

#### Publicly available datasets of RNAseq and microarray data

The Cancer Genome Atlas (TCGA) data portal 24 provides transcriptomic data on 287 colon adenocarcinoma samples and 24 matched normal tissues obtained by IlluminaHiSeq_RNASeqV2 technology (https://tcga-data.nci.nih.gov/tcga/). Transcript abundances were calculated in reads per kilobase of transcript per million mapped reads (RPKM).

Microarray data (GEO: GSE24550, deposited by Agesen *et al*. 25 were downloaded from the NCBI's Gene Expression Omnibus 26. The data were obtained from 77 CRCs and 13 normal colonic mucosa samples using the Affymetrix Human Exon 1.0 ST platform (Affymetrix, Santa Clara, CA, USA).

## Results

### Somatic point mutations of CHGs in colorectal tumors

**Figure 1:**
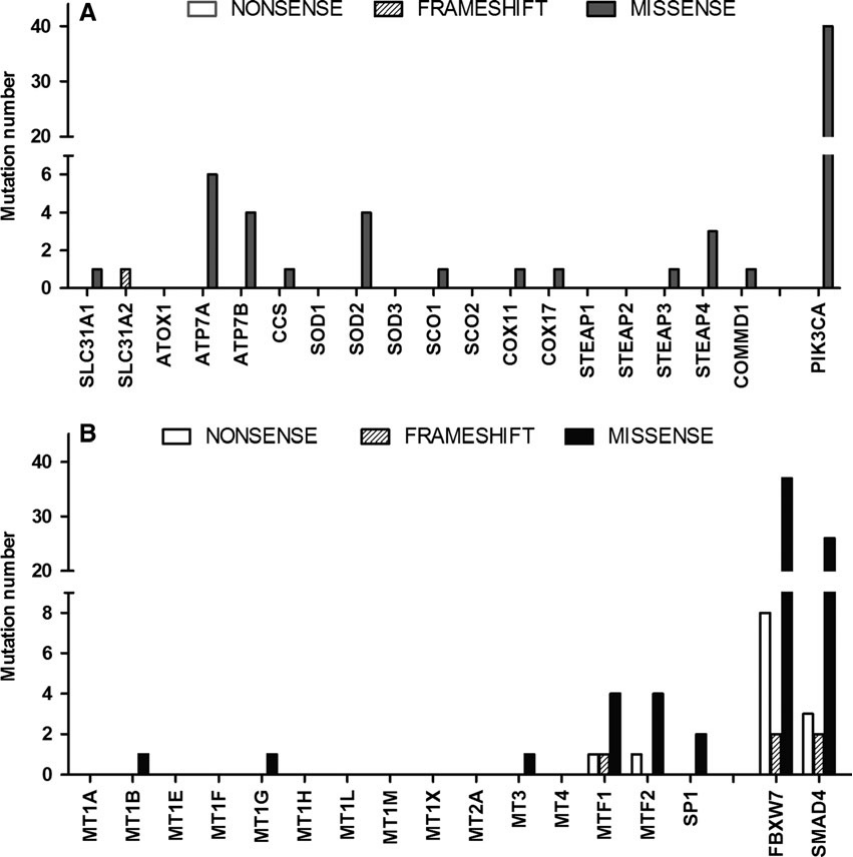
(A, B) Number of somatic point mutations (nonsense, frameshift, and missesnse) in coding sequences of CHGs detected by exome sequencing in 228 CRC tumors and normal pairs reported in the dataset published by The Cancer Genome Atlas.

In order to evaluate if inactivating point mutations of CHGs can be responsible for dysfunctions of copper homeostasis, The Cancer Genome Atlas Dataset (Cancer Genome Atlas Network, 2012) was analyzed. Data on point mutations detected by exome sequencing of 228 tumors and normal pairs are reported in such data set. In Fig. 1A,B the number of nonsense, frameshift, and missense mutations for CHGs are reported. The frequency of point mutations in CHGs is very low when compared with typical cancer gene drivers, such as ‘phosphatidylinositol‐4,5‐bisphosphate 3‐kinase, catalytic subunit alpha’ (PIK3CA). Therefore, these data do not support the hypothesis that accumulation of somatic mutations in CHGs can be responsible for a copper dyshomeostasis in CRC.

### mRNA levels of CHGs in colorectal tumors

**Figure 2:**
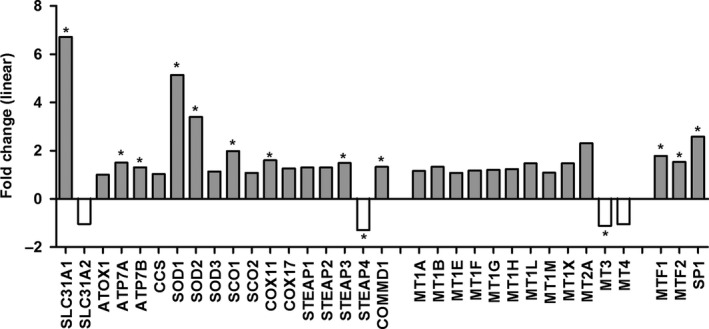
Differential expression of CHGs in CRC versus normal colonic mucosae (Mu). Values are expressed as linear fold changes calculated in the following way: 2^[^ ^CRC^ ^Average^ ^RMA^ ^– Mu average^ ^RMA^ ^]^ if CRC > Mu, or −2^[Mu Average^ ^RMA^ ^–^ ^CRC^ ^average^ ^RMA^ ^]^ if CRC < Mu. *False Discovery Rate (FDR) *P* < 0.002 and fold change > 1.25 or < −1.25.

**Figure 3:**
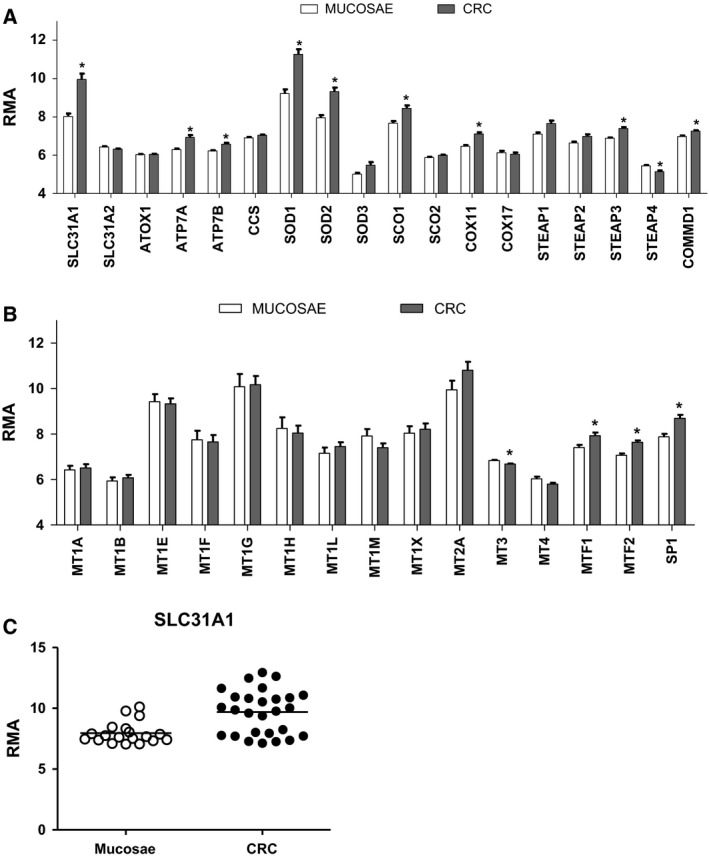
(A, B) Expression levels (RMA values) of the genes involved in copper homeostasis (CHGs) in 27 colorectal samples (CRC) and 19 matched normal colonic tissues. Statistical significance of comparison versus normal mucosae expressed as *FDR *P* < 0.002. (C) Scatter plot showing SLC31A1 RMA values in each colorectal sample and normal colonic mucosa.

As shown in Fig. 2 (linear fold changes) and Fig. 3A,C (RMA values) the mRNA for the copper transporter CTR1 (*SLC31A1* gene) is dramatically increased (6.72‐fold increase) in tumoral samples in comparison to normal colonic mucosae. Several other CHGs show a significant increase in tumoral samples: the efflux pumps ATP7A and B (1.5‐ and 1.31‐fold); the copper assembly factors SCO1 (1.98‐fold) and COX11 (1.6‐fold); the metalloreductase STEAP3 (1.49‐fold); the copper efflux protein COMMD1 (1.33‐fold); and the transcription factors MTF1, MTF2 (1.78‐ and 1.54‐fold), and SP1 (2.58). The cuproenzyme SOD1 (5.14‐fold) and the manganese enzyme SOD2 (3.4‐fold) are dramatically upregulated in CRC. Only STEAP4 shows a slight (−1.3‐fold) but statically significant decrease of expression in the CRC sample group in comparison to normal colonic mucosae.

**Figure 4:**
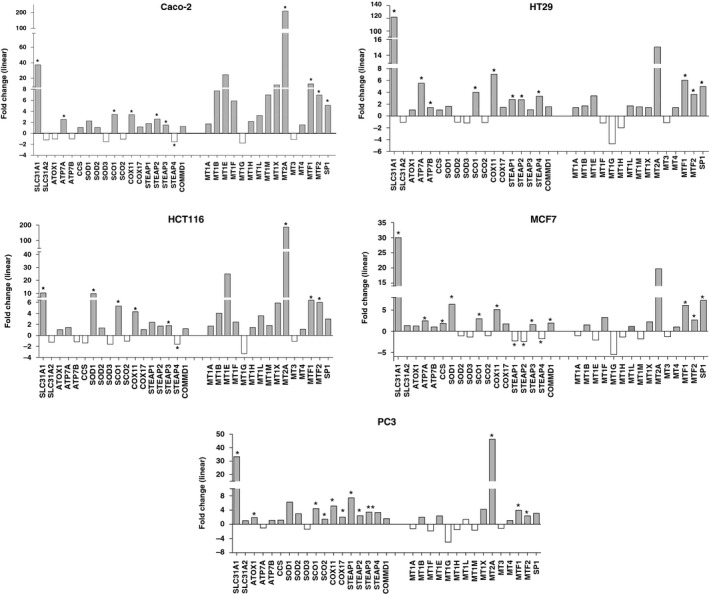
Linear fold changes are calculated in the following way: 2^[Cell Lines Average^ ^RMA^ ^– Mu average^ ^RMA^ ^]^ if cell lines > Mu, or −2^[Mu Average^ ^RMA^ ^– cell lines average^ ^RMA^ ^]^ if cell lines < Mu (*FDR *P* ≤ 0.005). Mu: normal colonic mucosae.

Similar changes are also detected in three colon cancer cell lines (Caco‐2, HT29, and HCT116) and in two cancer cell lines of different tissue origin (breast cancer cell line MCF7 and prostate cancer cell line PC3) (Fig. 4). In particular, the large increase in *SLC31A1*/CTR1 mRNA and the significant increase in SCO1 and COX11 mRNAs are present in all examined cell lines.

**Figure 5:**
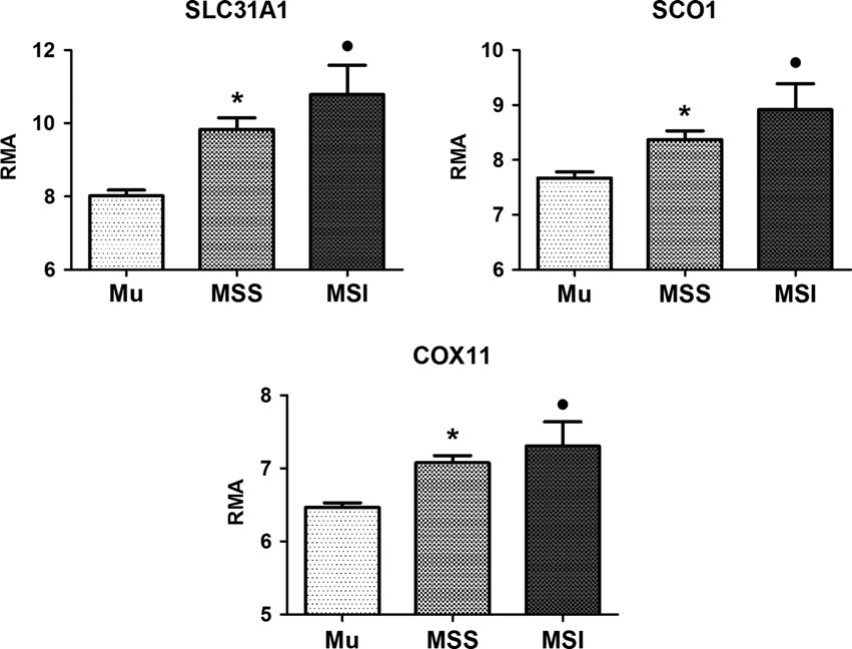
mRNA levels (RMA values) of SLC31A1/CTR1, SCO1, and COX11 genes in 22 MSS and 5 MSI colorectal tumors. In the same graph the RMA value of 19 matched normal colonic tissues is reported. Results are expressed as mean ± standard deviation. Statistical significance of comparison versus normal mucosae expressed as *, ^●^ FDR *P* < 0.002.

Based on a form of genetic instability, called microsatellite instability, CRC can be subdivided in two different classes; tumors with MSS and tumors with MSI. Microsatellites are tract of repetitive DNA formed by an individually fixed number of repeating units of 1–4 nucleotides. It is well‐known that a subgroup of CRC (10–15%) show a somatic variability in repeating units in cancer cells (MSI tumors). The presence of this form of genetic instability is associated with several biological feature of the tumor and with a better prognosis 23, 27. In our CRC samples, both MSS and MSI tumors show an increased expression of *SLC31A1*/CTR1, SCO1, and COX11 (Fig. 5). Values are slightly higher in MSI tumors but these differences are not statistically significant.

**Figure 6:**
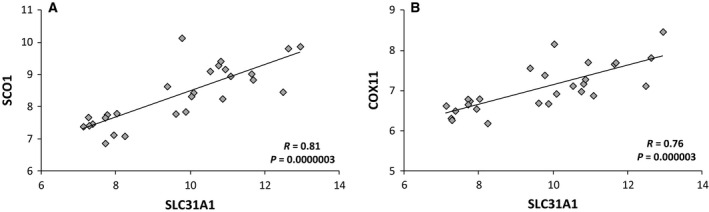
Correlation analysis between mRNA levels for the indicated genes. Pearson's correlation coefficients (R) are reported in each plot.

As shown in Fig. 6 a significant correlation between *SLC31A1* (encoding CTR1), *COX11*, and *SCO1* mRNA levels can be observed in CRC samples, suggesting that the transcriptional upregulation of these CHGs is part of a coordinated program of gene regulation.

### Exon‐level expression analysis of *SLC31A1* transcripts

In this study, transcript levels were determined by the Gene‐Chip Human Transcriptome Array 2.0 (Affymetrix). This high resolution array was designed with approximately ten probes per exon and four probes per exon–exon splice junction, thus allowing an analysis of expression at the exon level. The software ‘transcriptome analysis console’ (tac) 2.0 was used for the alternative splicing analysis using default parameters. The tac software provides a comparison of the expression of each exon (corresponding to a probe selection region, PSR) in two different conditions (in this work, CRC vs normal colonic mucosae). Such comparison is obtained by the so called ‘splicing index’: (intensity of exon X in CRC/intensity of gene Y in CRC)/(intensity of exon X in normal colonic mucosae/intensity of gene Y in normal colonic mucosae). Therefore, a positive index is obtained for exons prevalently expressed in cancer cells, while a negative one indicates a prevalent expression in normal colonic cells.

**Figure 7:**
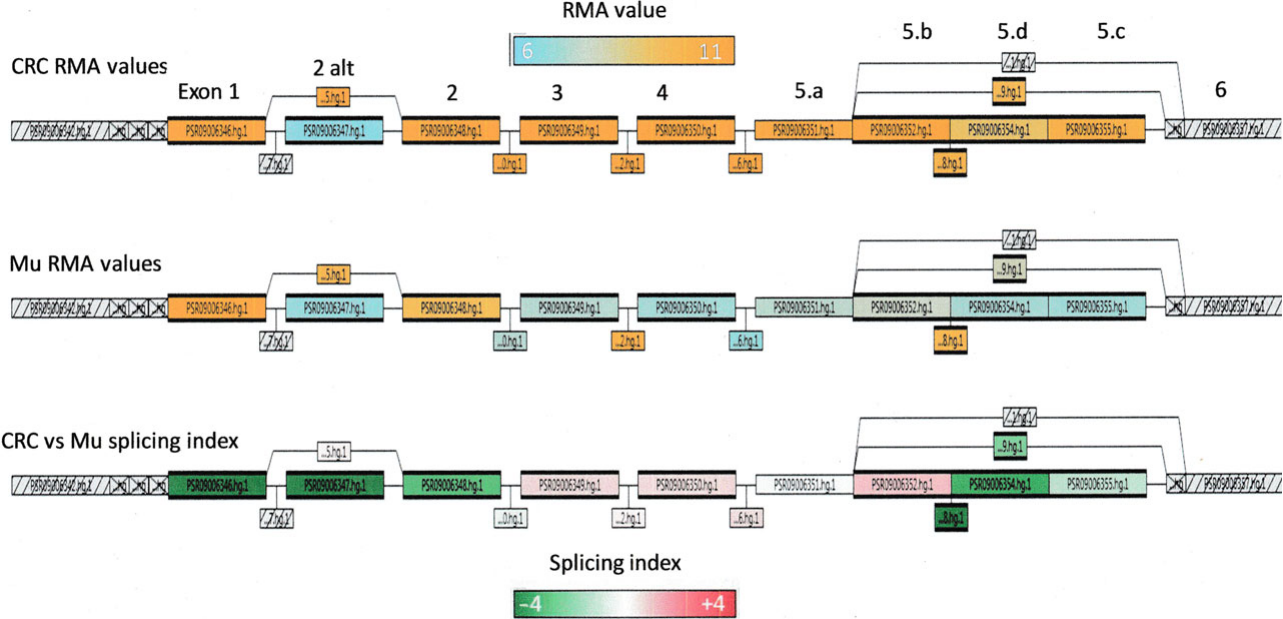
Gene Structure View of SLC31A1/CTR1 (tac software). All PSRs and Junctions are represented with boxes. The first two lines from the top display, in azure/orange scale, the RMA values for each PSR and corresponding exons: top line for CRC and middle line for normal mucosae (Mu). The bottom line displays the splicing index in a green/red scale.

**Figure 8:**
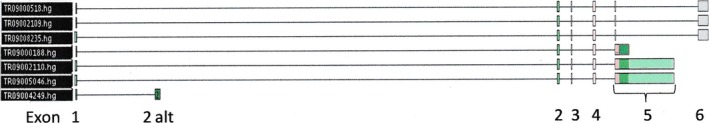
Alternative transcripts for SLC31A1/CTR1 on the basis of sequence data in public databases and in tac (Affymetrix) database.

The exon‐level analysis of *SLC31A1* is reported in Table 1A and in Fig. 7. The upregulation of *SLC31A1* involves the five exons (1, 2, 3, 4, and 5) that have been included in the classical structure of this gene (Fig. 8) 10, 28, 29. This concordance with the classical exon–intron structure of the gene is in favor of the synthesis of a functional protein in colorectal cancer. However, differences in the upregulation level of the different exons are also observed. The highest differences in RMA values between CRC and normal colonic mucosae (see also fold‐change values in Table 1A) are observed at the level of exon 3, 4, and 5b, and less differences are observed at the level of exon 1, 2, and 5d. The relatively high expression of exon 1 and 2 in the normal colonic mucosa might be explained by the expression of an alternative transcripts containing exon 1 and 2, but not exon 3 and 4, as also suggested by the significant negative splicing index (Table 1A, Fig. 7). This might be a functionally relevant observation as exon 2 encodes for the amino‐terminal part of the protein. On the contrary, the splicing index for exon 3, 4, and 5b is positive, a result that can be due to a prevalent expression of transcripts containing these exons in CRC.

**Table 1:** (A) Exon‐level analysis of SLC31A1/CTR1 gene: comparison between CRC samples and normal colonic mucosae (Mu). (B) Exon‐level analysis of SLC31A1/CTR1 gene: comparison between CRC cell lines and normal colonic mucosae (Mu)

Exon	PSR	mRNA/Protein	CRC RMA value	Mu RMA value	Fold change	Splicing Index	Splicing Index FDR
(A)							
1	PSR09006346.hg.1	5′ UTR	12.79	11.24	2.92	−5.18	0.000105
2alt	PSR09006347.hg.1	?	6.44	6.7	−1.2	−16.28	0.000001
2	PSR09006348.hg.1	5′UTR.CDS/aa 1‐43	11.32	9.43	3.72	−3.25	0.000764
3	PSR09006349.hg.1	CDS/aa 44‐68	12.57	7.24	40.26	1.52	0.041754
4	PSR09006350.hg.1	CDS/aa 69‐124	12.27	6.94	40.23	1.52	0.002176
5a	PSR09006351.hg.1	CDS/aa 125‐136	10.78	7.38	10.57	−1.10	0.561023
5b	PSR09006352.hg.1	CDS.3′UTR/aa 137‐190	13.04	7.77	38.58	1.80	0.000198
5d	PSR09006354.hg.1	3′ UTR	9.05	6.97	4.23	−3.48	0.001273
5c	PSR09006355.hg.1	3′ UTR	10.24	6.91	10.01	−1.88	0.003033
(B)							
1	PSR09006346.hg.1	5′ UTR	15.92	11.24	25.57	−6.61	0.000119
2alt	PSR09006347.hg.1	?	6.31	6.70	−1.31	−268.75	0.00000002
2	PSR09006348.hg.1	5′UTR. CDS/aa 1‐43	14.40	9.43	31.37	−5.76	0.00044
3	PSR09006349.hg.1	CDS/aa 44‐68	16.24	7.24	513.07	2.15	0.021
4	PSR09006350.hg.1	CDS/aa 69‐124	16.13	6.94	580.95	1.99	0.018
5a	PSR09006351.hg.1	CDS/aa 125‐136	14.28	7.38	119.71	−1.33	0.500
5b	PSR09006352.hg.1	CDS.3′UTR/aa 137‐190*	15.97	7.77	294.54	1.21	0.408
5d	PSR09006354.hg.1	3′ UTR	9.94	6.97	7.83	−12.78	0.000004
5c	PSR09006355.hg.1	3′ UTR	10.21	6.91	9.86	−5.51	0.000008

These results were confirmed by the comparison of cancer cell lines (Caco‐2, HT29, HCT116) versus the same set of normal colonic mucosae (Table 1B).

### Exon‐level expression analysis of *SLC31A1* transcripts in different publicly available datasets

**Figure 9:**
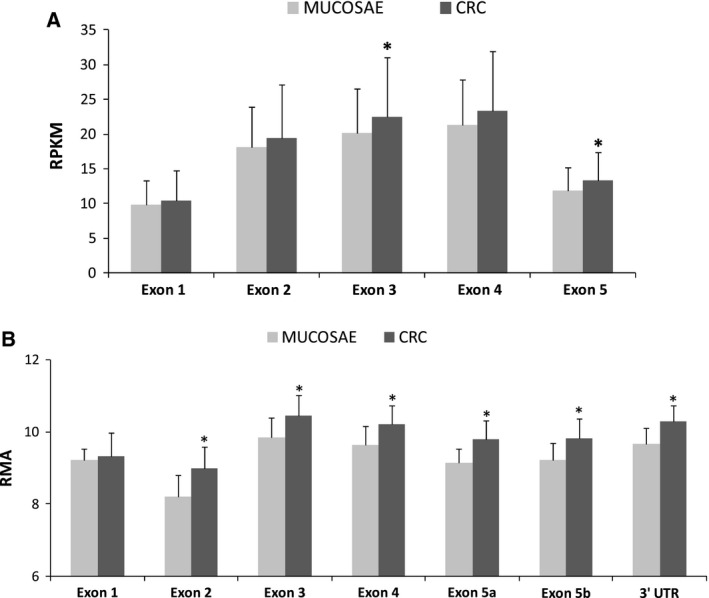
(A) RPKM values from RNA‐seq experiment. Data from TCGA relative to 287 colon cancer samples and 24 matched normal tissues. *P <* *0.05. (B) RMA values relative to different exons of SLC31A1 gene. Data from 77 CRCs and 13 normal colonic mucosa obtained by the Affymetrix Human Exon 1.0 ST platform (GEO: GSE24550). *P <* *0.05.

We also analyzed different publicly available datasets in order to confirm changes of *SLC31A1* gene expression in colorectal cancer. TCGA (2012) provides transcriptomic data on 287 colon cancer samples and 24 matched normal tissues obtained by RNA‐seq technology (https://tcga-data.nci.nih.gov/). A significant increase in RPKM values relative to exon 3 and 5 of *SLC31A1* can be detected (Fig. 9A). However, the fold‐increase values are much lower than those observed in HTA microarray.

In order to make a comparison with a more similar methodology, we downloaded expression microarray raw data from the Gene Expression Omnibus (GEO) public repository. In the series, GEO: GSE24550 25 exon‐level data from 77 CRCs and 13 normal colonic mucosa sample have been obtained by the Affymetrix Human Exon 1.0 ST platform. Data analysis of .cel files were performed using affymetrix^®^ expression console^™^ software v.1.4 and the affymetrix^®^ transcriptome analysis console (tac) software. As shown in Fig. 9B, a significant increase in RMA values was observed at the level of *SLC31A1* exon 2–5. In accordance with RNA‐seq data, the fold change increases (+ 40%) were lower than those observed in our HTA analysis. Such data are in agreement with an increase in *SLC31A1* transcript level, but dramatic differences in the fold‐change expression levels are revealed between HTA arrays and previous version of exon arrays or different methodologies, such as RNA sequencing.

## Discussion

Theoretically, accumulation of somatic mutations in cancer cells can modify a functional network with two different mechanisms: (a) a direct mechanism in which loss‐of‐function or gain‐of‐function somatic mutations affect genes whose products are mainly involved in a specific function, (b) an indirect mechanism in which somatic mutations take place in genes involved in signaling and regulatory factors able to induce alterations of multiple functional networks. Both types of mutations can be selected during the process of cancerogenesis if they are advantageous for tumor growth. With this in mind, in the present work, we try to answer some basic questions regarding mutational profile and gene expression of the functional network involved in copper trafficking and homeostasis pathways 4, 5 in colorectal cancer.

As a first step, we investigated the presence of somatic mutation in CHGs using the public database of somatic point mutations in CRC of The Cancer Genome Atlas Network (2012). Such analysis revealed that inactivating mutations are absent or extremely rare in genes encoding proteins involved in copper homeostasis. This observation suggests that inactivating point mutations are negatively selected during the cancerogenesis process since these proteins play a fundamental role in cell survival.

In a second step, we analyzed, at the mRNA level, the expression of CHGs in our series of colorectal carcinoma samples, showing an upregulation of several components of this functional pathway. In particular, we found a strong increase in mRNA levels of the copper transporter CTR1. Analysis of different publicly available gene expression datasets, obtained by RNA‐seq or previous version of Affymetrix exon microarray, confirmed a significant higher expression of *SLC31A1* transcripts in CRC, but revealed only weak changes compared to results obtained by HTA arrays. The reason for such quantitative difference is not clear at the moment and further research on gene expression normalization methods might shed light on this discrepancy.

CTR1 is the main Cu influx transporter in human cells and it has been shown that changes in its expression are able to induce modifications of cellular copper accumulation and resistance to copper cytotoxic effect 30, 31, 32. Unlike CTR1, the transcript for the low‐affinity copper transporter, CTR2, does not show any significant change in CRC. This observation is in agreement with the difference in function and regulation of expression between the two copper transporters 33. The observed CTR1 mRNA upregulation can be interpreted suggesting that cancer cells have a greater demand for copper than normal cells and that this requirement is linked to their proliferation and survival.

One of the limits of this investigation is the absence of data on CTR1 at the protein level. However, several studies show that transcriptional upregulation of the *SLC31A1* gene translates in higher protein levels and functional copper uptake 32, 34, 35. Moreover, an upregulation of CTR1 protein has been observed in a mouse model of HPV16‐induced cervical carcinoma. In this model, the CTR1 protein is highly expressed in tumors but not in the epithelium of the wild‐type cervix, the origin of cervical carcinoma 36. It is also relevant that CTR1 is required for ‘fibroblast growth factor’ (FGF), platelet‐derived growth factor (PDGF), and epidermal growth factor (EGF)‐induced mitogen‐activated protein kinase (MAPK) signaling 37 and that copper influx through CTR1 is required for oncogenic B‐Raf proto‐oncogene, serine/threonine kinase (BRAF) signaling and tumorigenesis 38, 39.

Few data regarding protein level of CTR1 in human cancer samples are available in the literature. Holzer *et al*. 40 have determined the pattern of CTR1 protein expression in normal and malignant human tissues using standard immunohistochemical techniques. No CTR1 expression was found in several common types of cancer, although metastatic colon carcinoma showed the highest level of expression among the malignant tissues 40. More recently Kim *et al*. 41 could detect the expression of CTR1 by immunohistochemistry in several samples of nonsmall cell lung cancer and corresponding normal epithelium.

A correlation between higher hCTR1 levels and higher platinum drug uptake in tumor cells sensitive to the drug has been reported 30, 31, 32, 36, 41. The observation that CTR1 expression is upregulated at similar levels in MSI and MSS tumors would suggest a sensitivity of both types of CRC to platinum drugs. Indeed, it has been reported that sensitivity to oxaliplatin was independent of the MSI status in CRC cell lines 42 and current clinical data suggest that MSI CRC may be sensitive to oxaliplatin 23.

The exon‐level comparison of *SLC31A1* transcripts in CRC and normal colonic mucosae suggests the possible existence of novel alternative transcripts and a different quantitative distribution of *SLC31A1* alternative transcripts in normal and cancer cells. Although the exact structure of novel alternative transcripts should be determined in future studies, these data are essential for the design of novel assays for the quantitative determination of CTR1 transcript level in cancer cells.

The increased *SLC31A1* mRNA level is accompanied by a parallel increase in transcript levels for copper efflux pump ATP7A, the copper assembly factor SCO1 and COX11, the cupric reductase STEAP3, and the transcription factors MTF1, MTF2, and Sp1. Such mRNA changes are highly correlated suggesting that they might be part of a coordinated transcriptional program. In this sense, the significant correlations between CTR1 (*SLC31A1* gene), SCO1, and COX11 mRNA levels suggest that such transcriptional upregulation might be part of a gene regulation program associated with cancer proliferation.

The transcriptional upregulation of copper transporters and chaperones in proliferating cells can be interpreted as a functional hyperactivity of copper trafficking pathways. Indeed, the observed transcriptional upregulation of the cuproenzyme *SOD* gene is coherent with an increase requirement and delivery of copper to intracellular sites. The observation that such coordinated transcriptional program is also conserved in the artificial growth conditions of the colon cancer cell lines suggests that it might represent a basic feature of proliferating cancer cells. This is also supported by the increased expression of *SLC31A1, SCO1*,* and COX11* mRNA observed in cell lines with a different tissue origin, such as MCF7 (breast cancer) and PC3 (prostate cancer).

A notable exception to the increased level of CHGs is represented by the copper chaperones ATOX1 and CCS. Although it is possible that other mechanisms of post‐translational regulation are operative for these proteins 43, 44, this aspect has not been further investigated in this study. Moreover, it has been reported that CTR1 silencing increases and copper supplementation decreases the protein level of ATOX1 and CCS 44. It is, therefore, conceivable that CTR1 overexpression in CRC is not accompanied by a parallel increase in ATOX1 and CCS mRNAs.

Our data are in agreement with previous results by Ishida *et al*. 45, suggesting that cancer cells might have a greater demand for and dependence upon copper. These researchers have observed increased levels of the copper transporter Ctr1 protein in mouse cervical carcinoma 36 and increased Ctr1 mRNA levels in islets undergoing tumorigenesis 45. Moreover, the same research group has shown that the activity of cytochrome c oxidase, a key enzyme in oxidative phosphorylation, in tumors is affected by copper chelating treatment 45. Interestingly, in the present work, we found a parallel transcriptional upregulation of CTR1 and the metallochaperones SCO1 and COX11 involved in the assembly of cytochrome c oxidase 46. COX11 and SCO1 are cometallochaperones assisting Cox17 in the copper insertion in cytochrome c oxidase. Copper metalation of the CuB site in the subunit 1 of cythocrome oxidase (Cox1) requires Cox11, while the metallochaperone SCO1 interacts with the subunit 2 of cytochrome oxidase (Cox2) to facilitate the maturation of its CuA site. It has been postulated that SCO1 mediates Cu(I) transfer from Cox17 to Cox2. Moreover, it has been recently shown a novel functional connection between SCO1 and CTR1; CTR1 is rapidly degraded in the absence of SCO1 protein, and its levels are restored in Sco1−/− mouse embryonic fibroblasts upon inhibition of the proteasome 47. Therefore, a functional synergy between CTR1 and SCO1 may meet the higher copper demand by CRC cells.

In conclusion, our results provide fundamental information on the transcript‐level expression of CHGs in colorectal cancer and further support the hypothesis that a subgroup of such cancers are characterized by a higher copper demand 45.
